# A Scoping Review Unveiling Antimicrobial Resistance Patterns in the Environment of Dairy Farms Across Asia

**DOI:** 10.3390/antibiotics14050436

**Published:** 2025-04-26

**Authors:** Yuvaneswary Veloo, Syahidiah Syed Abu Thahir, Zunita Zakaria, Salina Abdul Rahman, Rozaihan Mansor, Sakshaleni Rajendiran

**Affiliations:** 1 National Institutes of Health, Ministry of Health, Shah Alam 40170, Malaysia; syahidiah@moh.gov.my (S.S.A.T.); sar@moh.gov.my (S.A.R.); sakshaleni@moh.gov.my (S.R.); 2 Institute of Bioscience, Universiti Putra Malaysia, Serdang 43400, Malaysia; 3 Faculty Veterinary Medicine, Universiti Putra Malaysia, Serdang 43400, Malaysia; rozaihan@upm.edu.my

**Keywords:** antimicrobials, antimicrobial resistance, environment, Asia

## Abstract

Antimicrobial resistance (AMR) poses a significant “One Health” challenge in the farming industry attributed to antimicrobial misuse and overuse, affecting the health of humans, animals, and the environment. Recognizing the crucial role of the environment in facilitating the transmission of AMR is imperative for addressing this global health issue. Despite its urgency, there remains a notable gap in understanding resistance levels in the environment. This scoping review aims to consolidate and summarize available evidence of AMR prevalence and resistance genes in dairy farm settings. This study was conducted following the PRISMA Extension checklist to retrieve relevant studies conducted in Asian countries between 2013 and 2023. An electronic literature search involving PubMed, ScienceDirect, Embase, and Scopus resulted in a total of 1126 unique articles that were identified. After a full-text eligibility assessment, 39 studies were included in this review. The findings indicate that AMR studies in dairy farm environments have primarily focused on selective bacteria, especially *Escherichia coli* and other bacteria such as *Staphylococcus aureus*, *Klebsiella* spp., and *Salmonella* spp. Antimicrobial resistance patterns were reported across 24 studies involving 78 antimicrobials, which predominantly consisted of gentamicin (70.8%), ampicillin (58.3%), and tetracycline (58.3%). This review emphasizes the current state of AMR in the environmental aspects of dairy farms across Asia, highlighting significant gaps in regional coverage and bacterial species studied. It highlights the need for broader surveillance, integration with antimicrobial stewardship, and cross-sector collaboration to address AMR through a One Health approach.

## 1. Introduction

Milk and dairy products are known for their health benefits and are considered vital components of a well-balanced diet [1,2]. The nutrients from milk and dairy products are essential for most people, especially vulnerable populations, including infants, school children, and the elderly [1,2,3]. Bovine milk is the most frequently consumed dairy product worldwide, contributing significantly to global milk production [4]. According to the Food and Agriculture Organization (FAO), the production of milk has surged by 60% from 1987 to 2017, increasing from 522 million tons to 828 million tons [5]. FAO projections indicate a continued rise in global demand for milk and dairy products, with a particular focus on Asian countries [5].

Notably, Asia experienced the highest milk output expansion in 2018, surpassing other regions. According to the 2020 FAO dairy report, Asia recorded the largest increase in milk production, followed by Europe and other regions. In 2020, milk output reached 379 million tons, primarily driven by growth in India, China, and Pakistan [6]. This growth was largely attributed to an increase in dairy herd numbers and improvements in milk collection processes, which particularly benefited smallholder dairy producers [7].

The use of antimicrobials is a major concern for food safety and quality in the dairy industry [8]. The practice of administering antimicrobials in food animals contributed a significant portion of the total antimicrobial consumption. Antimicrobials are often used in livestock as growth promoters, prophylactics, and therapeutics. Consequently, these activities lead to environmental pollution with antimicrobials, antimicrobial-resistant bacteria, and antibiotic resistance genes (ARGs) [9,10].

The presence of these antimicrobial resistance (AMR) drivers amplifies the likelihood of interaction between antimicrobials and bacteria, fostering natural selection or mutation that favors resistance [11]. Subsequently, horizontal gene transfer may occur among environmental bacteria, leading to the dissemination of resistance, including pathogenic bacteria [12]. Humans, whether directly or indirectly, are exposed to these antimicrobial-resistant bacteria through food or the environment, thereby increasing the potential for infection [13].

A major global health burden, particularly in low- and middle-income countries (LMICs) is caused by AMR [14]. Based on studies on important pathogens including *Escherichia coli*, *Staphylococcus aureus*, and *Klebsiella pneumoniae*, it was predicted the continuous rise in AMR could kill 10 million people worldwide annually by 2050, especially in Asia and African countries [15]. Furthermore, resistance to last-resort antimicrobials garnered the attention of many authorities worldwide in mitigating it [16].

Asia is highly susceptible to the threats of AMR, and among its regions, Southeast Asian nations are assumed to be at the greatest risk of the emergence and spread of AMR [17]. This is largely attributed to the escalating use of antimicrobials in livestock production, particularly for growth promotion and treatment, which is further complicated by the easy access to antimicrobials and challenges in accessing quality veterinary care and preventive services [15,17]. The impact of AMR in Asia extends beyond clinical settings by increasing the burden of chronic diseases and widening health inequities [17]. These events culminate into a disproportionate AMR burden driven by imprudent antimicrobial stewardship practices and underdeveloped surveillance systems [14].

Global estimates have also shown that India and China are among the top five antimicrobial-consuming countries in terms of food-producing animals [4,14]. Despite the lack of comprehensive estimates, the veterinary sector accounts for 70% of antimicrobial consumption based on the surveillance of 36 commonly prescribed antimicrobials [17]. More importantly, three countries in the Southeast Asia region (Indonesia, Vietnam, and Myanmar) were among the five countries projected to record the largest increase in antimicrobial usage by food animals [18]. These projections highlight the risk of the emergence of AMR in Asia, likely to be driven by worsening antimicrobial usage hotspots and intensified farming systems.

Despite the burden of AMR in Asian countries, a review of the antimicrobial-resistant bacteria and ARGs, particularly in dairy farms and the immediate environment, is currently lacking. A recent systematic review of antimicrobial usage in animal production revealed that only 17 of the 89 articles reviewed were carried out in LMICs, with only two articles from the Southeast Asian region [8].

To elucidate the burden of antimicrobial-resistant bacteria in the environment, especially due to antimicrobial usage in dairy cattle, this scoping review was conducted to explore the burden of antimicrobial-resistant bacteria in dairy farm environments. The review aims to summarize the distribution of antimicrobial-resistant bacteria and ARGs in various dairy farms’ environmental samples focusing on Asian countries. Specifically, we aimed to present the available evidence addressing the following research questions: (i) What is the pattern of AMR in environmental bacteria on dairy farms? (ii) Which ARGs are highly present in the environment and among environmental bacteria? (iii) What are the various methods used to detect the presence of ARGs and their patterns on dairy farms?

## 2. Results

### 2.1. Selection of Evidence Source

**Figure 1:**
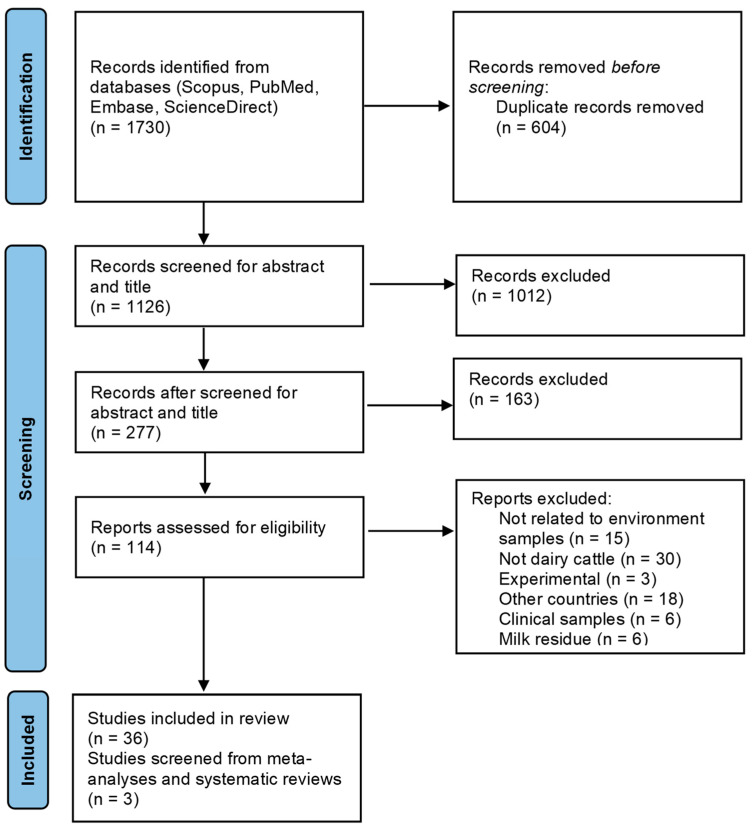
PRISMA flow chart of literature selection based on inclusion/exclusion criteria to identify the occurrence of AMR in the environment of dairy farms.

Our search strategy identified 1126 de-duplicated studies that underwent title and abstract screening. After initial screening, 114 studies were selected for full-text eligibility assessment, and of these, 78 studies were excluded due to the absence of environmental samples or because they entailed only milk residue, clinical, or experimental studies. Overall, a total of 39 studies were selected in this scoping review, with three additional studies identified through reference scanning. Figure 1 shows the Prisma flow diagram based on the PRISMA 2020 flow diagram with some modifications [19].

### 2.2. Overall Characteristics and Results of Sources of Evidence

**Figure 2:**
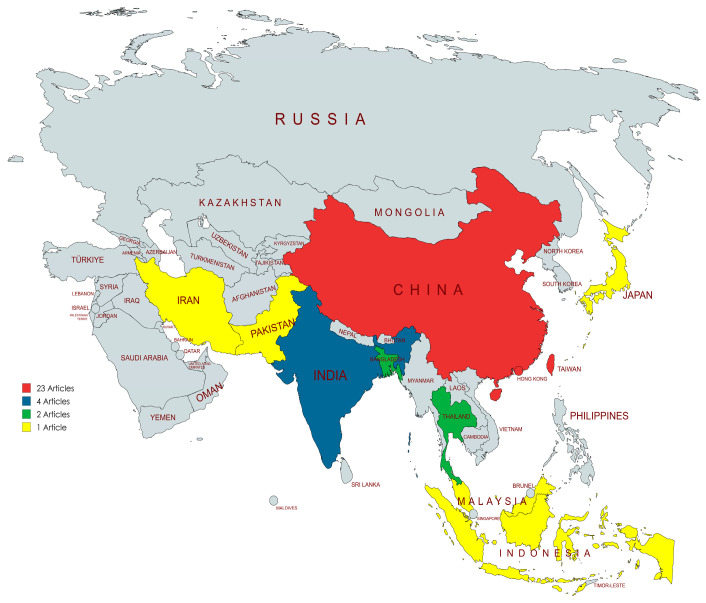
Asian countries with the number of articles. Map lines delineate study areas and do not necessarily depict accepted national boundaries.

Of the thirty-nine included studies, twenty-four (61.5%) were conducted in China, followed by India (four studies, 10.3%), Bangladesh (two studies, 5.1%), Thailand (two studies, 5.1%), and Indonesia (two studies, 5.1%). Single studies were conducted in Malaysia, Iran, Japan, Pakistan, and South Korea (Figure 2). Despite the presence of forty-eight countries and three dependencies in Asia, only 19.6% of these nations published studies related to AMR in the dairy farm environment. Most studies were published between 2019 and 2023 (69.2%; *n* = 27) compared to those published between 2013 and 2018 (30.8%; *n* = 12). The most published year was 2021 with nine (38.5%) articles.

The analyzed environmental samples comprised soil, water, manure, and effluent. Specifically, 26 studies (66.7%) included manure or feces as part of the environmental samples, 14 (35.9%) included soil, 14 (35.9%) focused on drinking or pipe water, and 10 (25.6%) considered effluent or wastewater. The summary of study characteristics is shown in Table 1.

### 2.3. Bacteria Analysis

Among the 39 studies included, 25 (64.1%) focused on the analysis of one or more bacteria. The most commonly studied bacteria were *E. coli* (*n* = 17, 68.0%), followed by *S. aureus* (*n* = 3, 12.0%), *Klebsiella* species (*n* = 2, 8.0%), and *Salmonella* species (*n* = 2, 8.0%). Table 2 summarizes the number of studies related to environmental bacteria, AMR testing methods, and ARGs. Notably, only one study analyzed bacterial presence but did not detect AMR, focusing instead on ARGs [20]. Several studies (*n* = 14, 35.9%) did not study the environmental bacteria as shown in Table 3.

### 2.4. Methods of Antimicrobial Susceptibility Testing

Various methods were used for antimicrobial susceptibility testing among the recovered isolates across studies, with the majority aligning with established standards such as CLSI, EUCAST, and others. Across the 24 studies, the predominant choice was the diffusion method (*n* = 15, 62.5%), followed by the dilution method to determine the minimum inhibitory concentration (MIC), either through broth medium (*n* = 8, 33.3%) or agar dilution (*n* = 1, 4.2%) (Table 2).

Overall, eight studies (33.3%) implemented agar supplemented with antimicrobials for screening purposes. One study reported the use of an E-test, a method that incorporates the principles of both dilution and diffusion approaches [59]. The usage of VITEK-2, an automated system, in two studies (8%) demonstrated the adoption of advanced technologies. Nevertheless, there is an opportunity for improvement, particularly in the accurate and rapid identification of bacterial resistance to antimicrobials [60]. Several studies did not state the methods of detection utilized for bacterial isolation and identification [23,32,40,41,42,43,47,50,52,53,54,58].

### 2.5. Antimicrobial Resistance Patterns

A total of 78 antimicrobials were used across 24 studies to assess resistance patterns among the isolates recovered from dairy farm environments in Asia. However, the number of antimicrobials tested by each study varied from one to twenty-six. Gentamicin appeared as the most frequently tested antimicrobial (*n* = 17, 70.8%), followed by ampicillin (*n* = 14, 58.3%), tetracycline (*n* = 14, 58.3%), ciprofloxacin (*n* = 13, 54.2%), chloramphenicol (*n* = 12, 50%), cefotaxime (*n* = 11, 45.8%), and trimethoprim-sulfamethoxazole (*n* = 11, 45.8%). Most studies reported high resistance to tetracycline, ampicillin, and trimethoprim-sulfamethoxazole compared to the rest [21,22,24,25,28,29,33,35,36,37,44,45,46,48,51,57] (Table 4).

Carbapenems, known for their broad-spectrum antibacterial activity, were tested in eleven studies on imipenem, nine studies on meropenem, and three studies on ertapenem, covering various bacteria. Most studies revealed high susceptibility (96.6–100%) to meropenem and imipenem, except for specific cases reporting resistance [22,28,29,33,36,39,44,45,46,48,56,57]. Aligning with the World Health Organization’s (WHO) priority list of antimicrobial-resistant bacteria [61], out of seventeen studies investigating *E. coli*, five reported extended-spectrum beta-lactamases (ESBL) isolates, exhibiting resistance to third-generation cephalosporins. Among the fecal samples, ESBL was reported at 66% (China), 47% (India), 25% (Indonesia), and 5.5% (Malaysia) [22,29,31,36].

Three studies focused on *S. aureus*, with one addressing methicillin-resistant *S. aureus* (MRSA) in 1.2% of environmental samples [34]. Two studies reported antimicrobial susceptibility testing for *Salmonella* species. One study reported the AMR rate in an aggregated manner, encompassing both environmental and milk samples [27]. Meanwhile, a 100% resistance to azithromycin was recorded among environmental samples, with higher resistance to erythromycin and tetracycline ranging from 83% to 93% across different samples within the environmental domain [46].

### 2.6. Antibiotic Resistance Genes (ARGs) Detection

Of the 32 studies (82.1%) involving ARG testing, 25 utilized polymerase chain reaction (PCR) testing, three concentrated on whole genome sequencing, and five employed metagenomic analysis. In line with antimicrobial detection preferences and findings, tet and sul genes were most commonly analyzed and found in abundance in the environmental samples [21,26,30,32,32,33,35,38,40,41,42,43,44,46,48,49] (Table 4).

Concerning *E. coli* strains, ESBL-associated genes were commonly tested, and blaTEM, blaSHV, and blaCTX were found in abundance [21,22,28,29,31,35,46,57]. Metagenomic sequencing, mainly used for the detection of ARGs in manure, soil, and wastewater, found that the predominant genes were tetracycline (tet), beta-lactams (blaTEM), sulfonamide (sul), and aminoglycoside genes [32,40,43,54,58]. ARGs were not explored in seven studies [24,25,27,36,37,45,58] as shown in Table 4. Most of the studies focused on gene detection [20,23,32,38,40,41,42,43,47,49,50,52,53,54,55,58], followed by those focusing on antimicrobial resistance testing [24,25,27,36,37,45,58] and those involving both aspects [21,22,28,29,30,31,33,34,35,39,44,46,48,51,57].

### 2.7. Comparative Analysis of AMR Patterns Across Environmental Samples Based on Country

Comparative analysis of AMR patterns was conducted across environmental samples in countries with at least two or more studies involving the same environmental sample.

China

Four studies in China assessed AMR patterns in fecal samples from dairy farm settings [24,32,38,58]. The diversity and abundance of ARGs in dairy feces were significantly higher compared to those detected in soil samples, with a high detection rate of tet(X) in feces at 71.4% [32,58].

Three studies focused on AMR patterns in manure samples. The positive detection rate for ARGs was higher than 80% in the studies (Wang et al., 2020; Wang et al., 2016; Yang et al., 2022) [49,50,53]. However, Yang et al. (2022) found that the abundance of the *bla* gene in dairy cattle feces was lower relative to the level in chicken and beef cattle farms [53].

As for wastewater, two studies recorded a significantly higher detection rate of ARGs in wastewater samples (100%) relative to soil samples, with the most abundant being sul1, sul2, tetM [22], and tet(X) [32]. Comparative analysis was not performed for feed, raw milk, and bedding samples, given that only one study was reported for each sample.

India

Among the four studies conducted in India, two involved cow dung/slurry [22,30], one involved soil samples [37] and one entailed floor and milking machine swabs [26]. However, all studies investigated the prevalence of *E. coli* and its resistant genes, except Gandhale et al. (2017) [26] who focused on *S. aureus*.

More than 50% of the isolates from cow dung/slurry were found to have resistant genes, especially tetA and sulII [30], while ESBLs were identified as the main cause of resistance to beta-lactam antimicrobials in *E. coli* [22].

Thailand

Two studies in Thailand also focused on AMR patterns in *E. coli*, particularly in water samples [28,39] and milk samples [39]. Both samples demonstrated resistant *E. coli* strains to ampicillin and carbenicillin, while *E. coli* from water samples possessed an ESBL phenotype and antimicrobial resistance *bla* genes [28,39].

Bangladesh

Two studies in this review explored AMR patterns from diverse environmental samples, including cow dung, water, feed, and soil, but the bacteria of interest differed between the studies, with *Listeria* spp. in one [45] and *E. coli* and *Salmonella* spp. in the other [46]. Comparative analysis was not feasible; nevertheless, the most predominant resistance gene identified in cow dung, soil, and water samples was *tetA* (80.5–84.7%) [46].

Indonesia

Two studies in Indonesia reported the AMR pattern of *E. coli* isolates from several environmental samples, including wastewater, feces, drinking water, milk, feed, and rinses of workers’ hands [25,36]. The occurrence of *E. coli* AMR ranged from 54% at the farm level to 99.1% at the subject level. The incidence of ESBL-producing *E. coli* was 22.8% in wastewater samples [25], whereas fecal samples recorded the highest positive results at 25% compared to the other samples [36].

### 2.8. Comparative Analysis of AMR Patterns Across Environmental Samples Between Countries

Comparative analysis between studies conducted in different countries was performed for only two environmental samples, fecal and wastewater, as they constituted the predominantly investigated samples in the reviewed studies.

Fecal samples

Fecal samples, including manure and cow dung, depicted consistent AMR patterns across the studies conducted in several countries, such as India, China, Bangladesh, Indonesia, and Japan [32,36,46,48,58]. The detection rate of ARGs, especially tetA in fecal samples, ranged from 71.4% to 84.7% [32,58], which was significantly higher compared to the detection rates in soil and water samples [46]. Resistant genes to *E. coli* were the predominantly investigated AMR pattern in the studies, with fecal samples recording significantly higher results for ESBLs, at 25% relative to soil and water samples [46].

In Japan, the highest frequency of antimicrobial-resistant *E. coli* strains isolated from environmental samples was from cow feces at 1.7%, with all strains resistant to tetracycline and possessing tetA [48]. Meanwhile, in Malaysia, fecal samples accounted for 5.5% of total samples positive for ESBL-producing *E. coli* [31].

Wastewater and drinking water samples

Consistent findings were observed in studies on AMR patterns in water samples, with resistant *E. coli* strains possessing an ESBL phenotype and antimicrobial resistance *bla* genes [28],38]. The incidence of ESBL-producing *E. coli* was 22.8% in wastewater samples [25], whereas wastewater and drinking water samples recorded the second and third-highest positive results at 16% and 10%, respectively [.

Likewise, wastewater samples depicted a significantly higher detection rate of ARGs compared to soil samples, with tet(A), tet(X), and tetM as the predominant resistance genes [23,29,32,46]. Suzuki et al. [48] also found an antimicrobial resistance rate of 8.3% (10/120) in the drainage from the studied dairy cattle barns, with the presence of tet(A) in all the tetracycline-resistant strains.

## 3. Discussion

Recognizing the environment as a crucial component of the “One Health” approach, continuous surveillance of this component is necessary, extending beyond the focus on human and animal health alone. This scoping review highlighted the research gap in the context of AMR within the environment domain in dairy farms around Asian countries. Despite dairy industries expanding in countries like India, China, Indonesia, the Philippines, Myanmar, Laos, and Vietnam, the majority of environmental AMR research is concentrated in China [62]. The lack of research highlights a crucial gap in understanding the extent and dynamics of AMR in dairy farm environments across broader Asian regions. Therefore, there is a need for comprehensive, regional studies to capture the full scope of AMR within these settings.

Most of the included studies focused on investigating the presence of AMR, specifically in *E. coli*, a bacterium that is commonly found in the intestinal tracts of mammals, including cows. The prominence of targeting *E. coli* in dairy farm environments can be attributed to its role as an indicator of fecal bacterium [63,64]. *E. coli* serves as a reliable indicator for addressing the AMR spread in various settings and is a common carrier of various ARGs [65]. However, this singular focus represents a major gap, as other significant pathogens, particularly the ESKAPE pathogens (*Enterococcus* spp., *Staphylococcus aureus*, *Klebsiella pneumoniae*, *Acinetobacter baumannii*, *Pseudomonas aeruginosa*, *and Enterobacter* spp.), remain largely studied.

These ESKAPE pathogens are identified by the WHO as critical MDR pathogens due to their ability to evade common antimicrobials, posing significant challenges in infection treatment and demanding the urgent development of new antimicrobials [66]. The scarcity of research on these pathogens in dairy farm environments limits the ability to assess their prevalence, transmission, and potential health risk to humans and animals [67,68,69]. Due to their potential for developing and extending resistance, future studies should focus on expanding surveillance including these high-risk pathogens.

The findings of this review reveal significant variations in resistance rates and the presence of ARGs, influenced by differences in the methods used for AMR testing and the types of samples analyzed. Despite these variations, a consistent trend emerges throughout the studies, which is the association between AMR and the use of antimicrobial agents for treatment on dairy farms [29,35,44,70]. The use of antimicrobials in dairy and food animals plays a significant role in shaping the AMR patterns in the farm environment. The frequent use of antimicrobials exerts selective pressure, favoring resistant strains that persist in environmental reservoirs such as soil and effluent, thereby contributing to the spread of resistant bacteria on farms [29,36,70]. Additionally, the genetic diversity of ARGs also plays a key role in influencing AMR through horizontal gene transfer [71,72].

For instance, the widespread emergence of colistin resistance is of particular concern, leading many countries in Asia to ban the use of colistin, especially in animal feeds, due to its importance as the last line of defense in human medicine. Interestingly, studies that have explored colistin resistance reported either total susceptibility or a very low level of resistance to this antimicrobial [33,44,45,51,57]. Although some studies reported low or no resistance to colistin, the detection of carbapenem-resistant bacteria from environmental sources raises concern regarding the use of antimicrobials in these areas. Carbapenem resistance poses a particularly severe threat to public health, due to their ability to facilitate horizontal gene transfer, further exacerbating AMR spread [73]. These findings underscore the urgent need for stringent antimicrobial stewardship practices and surveillance efforts to mitigate the spread of multidrug-resistant pathogens in dairy farm environments [74].

However, there were several limitations to this review, and these need to be acknowledged. The heterogeneity in study methodologies across the included papers, including differences in AMR susceptibility screening techniques, makes it difficult to compare across studies. Varying susceptibility testing methods, including disk diffusion, broth microdilution, and molecular approaches, can lead to different resistance profiles and hence, different study outcomes. Excluding gray literature and non-English articles, those published in Chinese may have introduced publication bias, thus limiting the scope of this review. Given that China has contributed significantly to AMR research, the omission of Chinese-language studies may lead to the underrepresentation of some important findings. Thus, future reviews should attempt to include a wider variety of studies in order to provide a more holistic assessment of AMR patterns in dairy farm environments. In addition, since this scoping review did not include a quality assessment and risk of bias analysis, it is recommended that further studies carry out a systematic review or meta-analysis to provide a more comprehensive and detailed investigation of AMR.

## 4. Materials and Methods

This review was performed using the methodological framework elaborated by Arksey and O’Malley and following the Prisma Extension for Scoping Review (PRISMA-ScR) checklist by Tricco et al. [75,76,77]. Therefore, only qualitative and comparative analyses were carried out in line with the provisions of a scoping review. Prior research has also shown that a scoping review encompasses studies with different research designs, which reflect a high degree of heterogeneity between the studies [77]. Thus, scoping reviews are exempted from robust quality assessment and statistical analyses.

### 4.1. Search Terms and Strategy

This review focused on AMR in environmental bacteria found in dairy farms. The study period covered research published between 2013 and 2023 to capture the latest pattern of AMR and ARGs in dairy farm environments. The review protocol was developed following the PRISMA-ScR guidelines, and no prior registration was conducted. Three reviewers (Y.V., S.R., and S.A.T.) independently performed a systematic bibliographic search across four databases—PubMed, ScienceDirect, Embase, and Scopus. The search strategy was initially drafted by Y.V. and subsequently reviewed and validated by S.R. and S.A.T. to ensure accuracy and consistency.

A comprehensive search of the relevant studies was conducted across 48 countries and 3 dependencies in Asia according to the United Nations [78] (Supplementary Table S1). The keywords used for the literature search were as follows: “cow”, “dairy”, “cattle”, “farm”, “farming”, “industry”, “environment”, “soil”, effluent”, “water”, “manure”, “wastewater”, “antibiotic”, “antimicrobial”, “resistant”, and “resistance”, and the general Boolean operators used for all four databases were as follows: (cow OR cows OR dairy OR cattle) AND (farm OR farms OR industry OR farming) AND (environment OR environments OR soil OR effluent OR water OR wastewater OR manure OR land) AND (antibiotics OR antibiotic OR antimicrobial OR antimicrobials OR multidrug OR resistant OR resistance). Country terms were not included in search terms, as studies conducted in Asia were filtered manually during the study selection phase. The search was conducted across the four mentioned databases. Subsequently, the records from each database were imported into Zotero reference management software version 6.0.37. Consequently, the software was used to remove duplicate entries of articles. All the records of the prospective articles were exported to a Microsoft Excel spreadsheet for study selection.

### 4.2. Eligibility Criteria

Independently, three reviewers (Y.V., S.R., and S.A.T.) completed the study selection based on the following inclusion criteria: (i) original, peer-reviewed, and primary research articles; (ii) studies addressing AMR in environmental bacteria found in dairy farm settings; (iii) published in English; (iv) published between 2013 and 2023; and (v) conducted in Asian countries (48 countries and 3 dependencies). Meanwhile, the exclusion criteria included (i) meta-analyses and all types of review articles; (ii) clinical, diagnostic, or health facility-based studies; and (iii) studies not involving environmental samples from dairy farms (e.g., studies focusing only on milk, dairy products, or human infections without environmental involvement). However, reference lists from these meta-analyses and systematic reviews were screened for additional relevant studies, leading to the inclusion of three additional articles.

### 4.3. Study Selection Process

A multi-step process was used to identify the studies. First, the title and abstract were screened based on the relevance to AMR in dairy farm environments in Asian countries. Next, full-text screening was conducted to ensure that the studies investigated AMR in environmental bacteria in dairy farms, were conducted in an Asian country, and reported the presence of AMR or ARGs. The study selection and screening process was performed independently by three reviewers (Y.V., S.R., and S.A.T.). Any disagreements were settled by discussion.

### 4.4. Data Management and Charting

All the data were extracted and charted using Microsoft Excel, including the study inclusion criteria and key variables. The data that were systematically extracted from each study included the author’s name, year of publication, study location, title of the research, research objectives, bacteria analyzed, types of environmental samples analyzed, methods used for AMR or ARG detection, antimicrobial agents tested, presence and percentage of resistance, ARG detected, and summary of key findings (Supplementary Table S2).

### 4.5. Data Extraction and Synthesis

Using a descriptive and narrative analysis style, specific data were collected from each study based on the subjects’ characteristics, types of environmental samples, country in which the study was conducted, methods used, and reported resistance patterns. Table 2 depicts the tested antimicrobials and ARGs based on their reported presence and percentage of resistance. Key findings were synthesized to identify trends, research gaps, and methodological limitations in the current literature on AMR in dairy farm environments.

## 5. Conclusions

This scoping review aimed to address the current status of AMR in the environmental domain of dairy farms across Asian regions. While AMR surveillance is an expanding area of research, significant knowledge gaps remain, particularly in regional coverage and limited focus on bacteria species. Thus, future studies should broaden geographical coverage and investigate emerging pathogens. Additionally, integrating environmental surveillance with antimicrobial stewardship is critical for mitigating the spread of resistance and protecting both human and animal health. Collaborative efforts among veterinary, agricultural, and public health sectors are essential in tackling AMR from a One Health perspective, ensuring sustainable dairy farming practices while safeguarding public health.

## Figures and Tables

**Table 1:** Summary of study characteristics (*n* = 39).

Characteristic	*n* (%)
Country	
China	24 (61.5)
India	4 (10.3)
Bangladesh	2 (5.1)
Thailand	2 (5.1)
Indonesia	2 (5.1)
Malaysia, Iran, Japan, Pakistan, South Korea	1 each (2.6)
Study period	
2023	3 (7.7)
2022	9 (38.5)
2021	7 (12.8)
2020	5 (17.9)
2019	3 (7.7)
2018	0
2017	2 (5.1)
2016	3 (7.7)
2015	3 (7.7)
2014	3 (7.7)
2013	1 (2.6)
Environmental samples	
Manure/feces	26 (66.7)
Soil	14 (35.9)
Drinking/piped water	14 (35.9)
Wastewater/effluent	10 (25.6%)
Feed	6 (15.4)
Floor	6 (15.4)
Bedding	2 (5.1)
Silage/compost	2 (5.1)
Milk equipment	2 (5.1)
Sink, fence, machine liner, dairy slurry	1 each (2.6)

**Table 2:** Types of environmental bacteria analyzed, AMR testing methods, and ARGs detection based on the number of studies involved.

Characteristics	*n* (%)
Type of bacteria analyzed (*n* = 25)	
*E. coli*	17 (68.0)
*S. aureus*	3 (12.0)
*Salmonella* spp.	3 (12.0)
*Klebsiella pneumoniae*	2 (8.0)
*Listeria monocytogenes*	2 (8.0)
*Bacillus cereus*, *Shigella* spp., *Shewanella* spp., *Acinetobacter portensis*, *Enterococcus* spp., *Stenotrophomonas maltophilia*	1 each (4.0)
AMR susceptibility testing methods (*n* = 24)	
Disk diffusion	15 (62.5)
Broth dilution (MIC)	8 (33.3)
Agar dilution	1 (4.2)
E-test	1 (4.2)
VITEK-2 system	2 (8.0)
ARGs detection (*n* = 32)	
Polymerase chain reaction	25 (78.1)
Whole genome sequencing	3 (9.4)
Metagenomic analysis	5 (15.6)

**Table 3:** The environmental matrix and characteristics of analysis conducted by the included studies.

Author, Year	Country	Year	Environmental Matrix	Isolation and Identification		Antimicrobial Susceptibility Testing		Antibiotic Resistance Genes	
				Species Studied	Methods of Detection	Method	Break-Points Used	Tested or Not	Method
Ali et al., 2021 [21]	Pakistan	2019	Wastewater	*E. coli*	Supplemented with colistin	Disk diffusion method or microdilution broth method	EUCAST, CLSI 2021, and CLSI 2020	Yes	Whole genome sequencing
Borah et al., 2014 [22]	India	2013	Cow dung	*E. coli*	Gram staining and biochemical analysis	Disk diffusion method	NCCLS, 2002	Yes	PCR and sequencing
Chen et al., 2015 [23]	China	2014	Wastewater and surface water	NA	NA	NA	NA	Yes	qPCR
Cui et al., 2016 [24]	China	2013–2014	Bedding. Feces, feed, liquid manure, and sink	*Bacillus cereus*	PCR	Microbroth dilution	CLSI	No	
Dameanti et al., 2023 [25]	Indonesia	NA	Wastewater	*E. coli*	Gram staining and biochemical analysis	Disk Diffusion Method	CLSI	No	
Gandhale et al., 2017 [26]	India	NA	Floor swab and milking machine	*S. aureus*	Biochemical analysis	Kirby–Bauer disk diffusion	CLSI	Yes	Multiplex PCR
Halimi et al., 2014 [27]	Iran	2009–2010	Milk filters, feeds, water, and milk fed	*Salmonella*	Biochemical analysis and PCR	Disk diffusion method	CLSI	No	
Hinthong et al., 2017 [28]	Thailand	NA	Water	*E. coli*	Gram staining, biochemical analysis, and PCR	Disk diffusion method	CLSI	Yes	PCR
Huang et al., 2022 [29]	China	2020–2021	Cow feces, sewage, fence, sink, soil, and feed	*E. coli*	Biochemical analysis, supplemented with cefotaxime, and PCR	Double disk diffusion method (ESBL) and micro-agar dilution method	CLSI	Yes	PCR
Jindal et al., 2021 [30]	India	NA	Slurry, animal drinking water, and pond water	*E. coli* and *Klebsiella*	Biochemical analysis	Disk diffusion method	NA	Yes	Multiplex PCR
Kamaruzaman et al., 2020 [31]	Malaysia	NA	Drinking water, source of drinking water, feed, house flies, floor, feed, and water troughs	*E. coli*	Biochemical analysis and PCR	Double-disk diffusion method	NA	Yes	Multiplex PCR
Kang et al., 2022 [32]	China	2017–2018	Feces, wastewater, and soil	NA	NA	NA	NA	Yes	Metagenomic sequencing
Li et al., 2022 [33]	China	2021	Feces, milking environment, and shed environment	*Acinetobacter portensis*, *Shewanella* spp., and *Stenotrophomonas maltophilia*	Supplemented with meropenem and 16s-rRNA gene sequencing	Broth microdilution method	CLSI and EUCAST	Yes	PCR and whole genome sequencing
Lim et al., 2013 [34]	Korea	2008	Milk cup, floor, fence, ventilation fan, water, and feed	*S. aureus*	Supplemented with cefoxitin and PCR	Disk diffusion method AND E-test strips	CLSI	Yes	Multiplex PCR
Liu et al., 2021 [35]	China	NA	Wastewater	*E. coli*	Microbial mass spectrometry identification	MIC method	CLSI	Yes	PCR
Maulana et al., 2021 [36]	Indonesia	2020	Wastewater, drinking water, and feed	*E. coli*	Biochemical analysis, supplemented with cefotaxime	VITEK-2 system	CLSI, EUCAST, and Global European	No	
Parul et al., 2014 [37]	India	2012–2013	Soil	*E. coli*	Biochemical analysis	Disk diffusion method	CLSI	No	
Peng et al., 2022 [38]	China	NA	Feces	NA	PCR	NA	NA	Yes	qPCR
Pumipuntu and Pumipuntu, 2020 [39]	Thailand	NA	Water	*E. coli*	Biochemical analysis	Disk diffusion method	CLSI	Yes	PCR
Qi et al., 2023 [40]	China	2022	Water, manure, feed, and soil	NA	NA	NA	NA	Yes	qPCR and metagenomic sequencing
Qi et al., 2022 [41]	China	2019	Soil	NA	NA	NA	NA	Yes	qPCR
Qi et al., 2021 [42]	China	NA	Soil	NA	NA	NA	NA	Yes	qPCR
Qiu et al., 2022 [43]	China	2017–2018	Manure and compost	NA	NA	NA	NA	Yes	Metagenomic sequencing
Shoaib et al., 2023 [44]	China	2017–2019	Feces, manure slurry, water, soil, and crop field soil	*E. coli*	VITEK-2 system and PCR	Micro-dilution assay	EUCAST	Yes	PCR
Shourav et al., 2020 [45]	Bangladesh	2018–2019	Cow dung, water, and feed	*Listeria* spp.	Biochemical analysis	Kirby–Bauer disk diffusion	CLSI	No	
Sobur et al., 2019 [46]	Bangladesh	NA	Cow dung, soil, and water	*E. coli* and *Salmonella*	Biochemical analysis and PCR	Disk diffusion method	CLSI	Yes	PCR
Sun et al., 2015 [47]	China	2014	Soil	NA	NA	NA	NA	Yes	qPCR
Suzuki et al., 2022 [48]	Japan	2018–2019	Feces, drainage, and wastewater	*E. coli*	MALDI-TOF-MS	Microliquid dilution method	CLSI	Yes	PCR
Wang et al., 2016 [49]	China	2014	Manure, amended soil, water, surface water, and wastewater	NA	NA	NA	NA	Yes	qPCR
Wang et al., 2020 [50]	China	NA	Manure	NA	NA	NA	NA	Yes	RT-qPCR
Wu et al., 2022 [51]	China	2019–2020	Bedding, feed, feces, air, drinking water, spraying water, washing water, and milk cup	*Klebsiella*	Biochemical analysis and PCR	Microbroth dilution method	CLSI, EUCAST	Yes	PCR, sanger sequencing
Xi et al., 2015 [20]	China	NA	Fresh cow pats, wastewater, surface water, soil, river water, and drinking water	*E. coli*, *Enterococcus*, *S. aureus*, *Shigella*, and *Salmonella*	PCR	NA	NA	Yes	qPCR
Yang et al., 2021 [52]	China	2019	Feces, solid waste, wastewater, and soil	NA	NA	NA	NA	Yes	PCR, qPCR
Yang et al., 2022 [53]	China		Manure and cowshed Wastewater	NA	NA	NA	NA	Yes	qPCR
Zhang et al., 2020 [54]	China	2017	Feces and manure	NA	NA	NA	NA	Yes	Metagenomic sequencing
Zhang et al., 2019 [55]	China	2015–2016	Feces	*E. coli*	Supplemented with sulfate colistin and PCR	NA	NA	Yes	PCR
Zhao et al., 2021 [56]	China	2018–2019	Feces, hide swabs, silage, and drinking water	*Listeria*	PCR	Disk diffusion method	CLSI	No	
Zheng et al., 2019 [57]	China	2016	Feces	*E. coli*	MALDI-TOF MS	VITEK-2	CLSI	Yes	Whole genome sequencing
Zhou et al., 2016 [58]	China	NA	Feces and soil	NA	NA	NA	NA	Yes	qPCR and metagenomic sequencing

NA = not applicable.

**Table 4:** Major findings of the 39 included studies on AMR and ARGS in the environment of dairy farms.

Author, Year	Focus	Highlights of the Findings
Ali et al., 2021 [21]	AR and GD	*E. coli* PK-3225 carried colistin, beta-lactam, tetracycline, and other resistance genes.
Borah et al., 2014 [22]	AR and GD	ESBLs were the main cause of resistance in *E. coli*. In total, 73.75% of isolates were resistant to at least one of the 3rd generation cephalosporins. Both blaTEM and blaSHV were found in 21.42% of the isolates.
Chen et al., 2015 [23]	GD	A total of 22 ARGs detected in samples, with sul1, sul2, and tetM being the most abundant, with a frequency of 100%. The abundance of ARGs in wastewater is high.
Cui et al., 2016 [24]	AR	Antimicrobial resistance was common in *B. cereus*-like isolates. *B. cereus* group strains were sensitive to certain antibiotics (ciprofloxacin, gentamicin, linezolid, streptomycin, and virginiamycin), while resistant to others (lincomycin, retapamulin, tiamulin, and valnemulin).
Dameanti et al., 2023 [25]	AR	In total, 99.17% of *E. coli* isolates showed antimicrobial resistance, and 84.25% of *E. coli* isolates were multidrug-resistant. ESBL-producing *E. coli* incidence in wastewater was 22.80%.
Gandhale et al., 2017 [26]	AR and GD	*S. aureus* isolates were sensitive to netilmicin, amikacin, tobramycin, and gentamicin, and resistant to penicillin, kanamycin, and oxacillin. Antimicrobial resistant genes blaZ, ermB, and tetK were detected in isolates.
Halimi et al., 2014 [27]	AR	Eighteen out of nineteen *Salmonella* spp. were resistant to oxytetracycline. A total of 26.3% showed resistance to more than one antibiotic. Enrofloxacin was the most susceptible antibiotic against all isolates.
Hinthong et al., 2017 [28]	AR and GD	*E. coli* from water samples showed resistance to ampicillin and carbenicillin and carried ESBL phenotype and ARGs (blaTEM and blaCMY-2). In total, 24 *E. coli* isolates showed positive results for virulence gene detection.
Huang et al., 2022 [29]	AR and GD	Overall, 19.5% of ESBL *E. coli* were isolated from five dairy farms. In total, 91.3% of strains were resistant to three or more antibiotics. blaCTX-M1 was the most dominant gene followed by blaCTX-M9 and aadA1.
Jindal et al., 2021 [30]	AR and GD	*E. coli* and *Klebsiella* showed resistance genes in various samples. ARGs were most prevalent in slurry with 193 genes. TetA was the highest followed by sulII and qnrS.
Kamaruzaman et al., 2020 [31]	AR and GD	A total of 4.8% of samples had ESBL-producing *E. coli*, mainly in milk. Predominant ESBL genotypes were a combination of TEM and CTX-M. There was no association between ESBL-producing *E. coli* in lactating cows and milk.
Kang et al., 2022 [32]	GD	ARGs resistant to tetracyclines, aminoglycoside, β-lactams, and MLS were dominant, and a high abundance of tet(X) was found in wastewater and feces.
Li et al., 2022 [33]	AR and GD	*Shewanella* spp.-carrying blaNDM-1 and *Acinetobacter portensis*-harboring tetX3 and blaNDM-1 were found.
Lim et al., 2013 [34]	AR and GD	In total, 1.2% of environmental samples were MRSA-positive. MRSA isolated from environmental samples was genetically identical to that from milk isolates.
Liu et al., 2021 [35]	AR and GD	*E. coli* isolates were mainly resistant to β-lactams and tetracyclines. TetA gene was highly found, with limited dissemination of sulphonamide-resistance genes in the study area.
Maulana et al., 2021 [36]	AR	Overall, 54% were ESBL-producing *E. coli* and 50% of them were multidrug resistant. Resistance to trimethoprim, tetracycline, and gentamicin was observed in isolates.
Parul et al., 2014 [37]	AR	The majority of the *E. coli* isolates were highly susceptible to ceftriaxone, amikacin, ciprofloxacin, and gentamicin but highly resistant to amoxicillin and tetracycline.
Peng et al., 2022 [38]	GD	A relative abundance of ARGs presenting resistance to tetracycline and aminoglycoside was observed, with tetM, cmx(A), sul1, tetW, ermB, and qacE∆1–01 being the most abundant.
Pumipuntu and Pumipuntu, 2020 [39]	AR and GD	A total of 1.43% of *E. coli* resistant to carbapenem in were found Saraburi and two isolates resistant to imipenem in Kaeng Khoi, while no drug-resistant *E. coli* were found in Sarakham. The blaNDM gene was detected in drug-resistant *E. coli* isolates.
Qi, 2023 [40]	GD	High abundance of β-lactamase resistance genes with blaTEM content as high as 94.55% among all ARGs was observed.
Qi et al., 2022 [41]	GD	The abundance of β-lactam ARGs increased with tetracycline and β-lactams. It was noticed that tetracycline ARGs remained stable with increasing antibiotic concentrations, while sulfonamide ARGs decreased with rising antibiotic levels.
Qi et al., 2021 [42]	GD	Significant correlations between heavy metals and ARGs in soil were found. The top ARGs were sul2, tetX, and blaTEM.
Qiu et al., 2022 [43]	GD	In total, 201 ARGs were shared in different animal manures and reported for 86–99% of total abundance. Compost had a significantly lower abundance of ARGs compared to manure.
Shoaib et al., 2023 [44]	AR and GD	Most *E. coli* isolates were resistant to sulfamethoxazole/trimethoprim, cefotaxime, ampicillin, ciprofloxacin, and tetracycline. All isolates were susceptible to meropenem, tigecycline, and colistin sulfate. A total of 44% showed multidrug resistance, and 90% of ARGs were detected.
Shourav et al., 2020 [45]	AR	The prevalence of *Listeria* spp. was 13.2% in cattle farm environments. *Listeria* spp. isolates showed resistance to multiple antibiotics with at least eight antibiotic classes.
Sobur et al., 2019 [46]	AR and GD	*E. coli* and *Salmonella* spp. were highly resistant to specific antibiotics and showed resistance to multiple antibiotics. ARGs ereA, tetA, tetB, and SHV were detected with tetA being the highest and SHV being the lowest.
Sun et al., 2015 [47]	GD	The sulI, sulII, ermA, and ermB were 100% detected in the soil samples. There was a strong positive relationship between ARG abundance and bio-accessible OP content.
Suzuki et al., 2022 [48]	AR and GD	A total of 1.7% of cow feces strains were resistant to tetracycline, all other strains were susceptible. The drainage resistance rate 8.3%, and all strains showed susceptibility for 8 months. Tetracycline resistance is common in animal feces and water samples. The TetA gene was detected in all strains.
Wang et al., 2016 [49]	GD	The spread of sul genes was strong and extensive. A higher abundance of tet genes expressing ribosomal protection proteins and ermB genes was found. Most ARGs showed significant positive relationships with environmental variables.
Wang et al., 2020 [50]	GD	Overall, 89.17% of ARGs were detected in manure. Tiamulin was identified to have a significant correlation with optrA, which was the most abundant.

AR = antibiotic resistance testing. GD = gene detection. AMR = antimicrobial resistance. ARGs = antibiotic resistance genes. ESBL = extended-spectrum beta-lactamase. *E. coli* = *Escherichia coli*. *B. cereus* = *Bacillus cereus*. *S. aureus = Staphylococcus aureus.* spp. *= species.* MRSA = methicillin-resistant *Staphylococcus aureus*. *K. pneumoniae* = *Klebsiella pneumoniae*. *L. monocytogenes* = *Listeria monocytogenes*.

## Data Availability

The data included in this review are referenced in this article.

## Supplementary Materials

The following supporting information can be downloaded at: https://www.mdpi.com/article/10.3390/antibiotics14050436/s1, Table S1: List of countries in the Asian Region; Table S2: The details on the included articles.

## Abbreviations

The following abbreviations are used in this manuscript:

AMR	Antimicrobial resistance
FAO	Food and Agriculture Organization
ARGs	Antibiotic resistance genes
LMICs	Low- and middle-income countries
*E. coli*	*Escherichia coli*
*S. aureus*	*Staphylococcus aureus*
MIC	Minimum inhibitory concentration
WHO	World Health Organization
ESBL	Extended-spectrum beta-lactamases
PRISMA-ScR	Prisma Extension for Scoping Review
