# Supplementary material for: A Scoping Review Unveiling Antimicrobial Resistance Patterns in the Environment of Dairy Farms Across Asia
Source: Antibiotics (Basel). 2025 Apr 26;14(5):436. doi: 10.3390/antibiotics14050436 (PMC12108256; doi:10.3390/antibiotics14050436)
Supplement: Supplementary file 1 [file antibiotics-14-00436-s001.zip › Table S1.pdf]

Table S1. List of countries in the Asian Region

No.	Country	Subregion
1	India	Southern Asia
2	China	Eastern Asia
3	Indonesia	South-Eastern Asia
4	Pakistan	Southern Asia
5	Bangladesh	Southern Asia
6	Japan	Eastern Asia
7	Philippines	South-Eastern Asia
8	Vietnam	South-Eastern Asia
9	Iran	Southern Asia
10	Turkey	Western Asia
11	Thailand	South-Eastern Asia
12	Myanmar	South-Eastern Asia
13	South Korea	Eastern Asia
14	Iraq	Western Asia
15	Afghanistan	Southern Asia
16	Yemen	Western Asia
17	Uzbekistan	Central Asia
18	Malaysia	South-Eastern Asia
19	Saudi Arabia	Western Asia
20	Nepal	Southern Asia
21	North Korea	Eastern Asia
22	Syria	Western Asia
23	Sri Lanka	Southern Asia
24	Kazakhstan	Central Asia
25	Cambodia	South-Eastern Asia
26	Jordan	Western Asia
27	United Arab Emirates	Western Asia
28	Tajikistan	Central Asia
29	Azerbaijan	Western Asia
30	Israel	Western Asia
31	Laos	South-Eastern Asia
32	Turkmenistan	Central Asia
33	Kyrgyzstan	Central Asia
34	Singapore	South-Eastern Asia
35	Lebanon	Western Asia
36	State of Palestine	Western Asia
37	Oman	Western Asia
38	Kuwait	Western Asia
39	Georgia	Western Asia
40	Mongolia	Eastern Asia
41	Qatar	Western Asia
42	Armenia	Western Asia
43	Bahrain	Western Asia
44	Timor-Leste	South-Eastern Asia
45	Cyprus	Western Asia
46	Bhutan	Southern Asia
47	Maldives	Southern Asia
48	Brunei	South-Eastern Asia

Dependencies or other territories

No.	Territory	Dependency of
1	Taiwan	China
2	Hong Kong	China
3	Macao	China

Source from https://www.worldometers.info/geography/how-many-countries-in-asia/
